# Isolation, social support, and COVID-19-burnout among college students in a university in eastern China

**DOI:** 10.3389/fpsyg.2025.1576596

**Published:** 2025-08-20

**Authors:** Yan Song, Huizi Li, Yuxin Song, Gexuan Song, Qing Su, Na Liu, Zheng Zheng, Yueyi Sun

**Affiliations:** ^1^Department of Psychology, School of Medicine, Nanjing University of Chinese Medicine, Nanjing, China; ^2^Department of Geriatric Psychology, School of Elderly Care Services and Management/School of Aging Industry, Nanjing University of Chinese Medicine, Nanjing, China

**Keywords:** COVID-19-burnout, social support, isolation, college student, mental health

## Abstract

**Background:**

To cope with the COVID-19, social isolation applied by countries around the world. These measures have significantly impacted public health, particularly among Chinese college students. Considering the significant impact of social isolation on social support, and the important value of social support for mental health, this study aims to explore the effects of social isolation on the mental health of Chinese college students during the pandemic.

**Methods:**

The Social Support Scale, COVID-19-burnout Scale Questionnaire, and Brief Symptom Checklist were used in this survey. In November 2022, a total of 394 college students voluntarily participated in this survey. And 70.3% of them were female, mean age = 19.09 ± 0.82 years), with 31.0% (*n* = 122) isolated and 69.0% (*n* = 272) non-isolated.

**Results:**

(1) Compared with the non-isolated group, the isolated group of college students scored significantly lower on social support (t = −3.40, *p* < 0.01), higher on COVID-19-burnout (t = 2.42, *p* < 0.01) and psychological distress (t = 2.96, *p* < 0.01); (2) Significant negative correlations emerged between social support and COVID-19-burnout (r = −0.142, *p* < 0.01), as well as psychological distress (r = −0.356, *p* < 0.01); (3) Within-family social support correlated negatively only with psychological distress (r = −0.314, *p* < 0.01), whereas outside-family social support was negatively associated with both COVID-19-burnout (r = −0.157, *p* < 0.01) and psychological distress (r = −0.339, *p* < 0.01). (4) Isolation moderated the relationship between social support and COVID-19-burnout (β = −0.213, *p* < 0.01; interaction β = 0.198, *p* < 0.01), but not social support and psychological symptoms (*p* = 0.26).

**Conclusion:**

This study found the negative impacts of social isolation and positive influence of social support during COVID-19 on the mental health and COVID-19-burnout of college students. It also highlights the influence of extra-family social support in reducing psychological distress and COVID-19-burnout. And isolation was found acted as a significant variable between social support and COVID-19-burnout. These results provide a new insight for understanding the mechanism through which social support impacts the mental health, and suggesting tailored interventions for Chinese college students in the context of COVID-19. The cultural characteristics during the isolation process and some limitations of this research were discussed also.

## 1 Introduction

### 1.1 Social support and isolation

It is well-known that social support plays an important role in an individual’s health (Tajvar et al., 2008). The presence of a supportive social network is instrumental in enabling individuals to fully exploit the advantages of positive life experiences and accomplishments, thereby highlighting the indispensability of social relationships in improving overall life satisfaction (Gable and Bedrov, 2022). It is characterized as the support an individual obtains through a complex web of social interactions involving other individuals, various groups, and the overarching community (House et al., 1988).

On the other hand, social isolation is thought to reduce the role of social support and thereby raise the risk to mental health. Social support has been severely curtailed due to the reduced contact between people and social isolation that occurred subsequent to the outbreak of COVID-19 (Grey et al., 2020). Individuals who are socially isolated are more likely to have a higher risk of experiencing negative mental and physical health outcomes, such as anxiety and depression (Evans and Fisher, 2022).

Since COVID-19 pandemic, strict quarantine measures were included to control the spreading of it in China. Evidence suggests that these isolation policies have increased mental health risks among residents, including higher rates of depressive symptoms, psychological distress (Zhong et al., 2020), and loneliness (Xu et al., 2023). The same results were found in college student ether. It was found that lockdown of university dormitories during the pandemic negatively impacted college students’ health, evidenced by shifts in their lifestyle behaviors, exercise habits, and sleep patterns (Zhang et al., 2022). Several maladaptive coping strategies, such as denial, substance use, and behavioral disengagement, were found to increase in college students during this difficult time (Samuolis et al., 2023).

During this hard period, it was suggested that social support could help college students ease psychological stress and strengthened resilience by offering emotional comfort and practical help (Browning et al., 2021), cope with academic challenges and life stressors (Duan and He, 2023), improves the emotional state and well-being (Lopez et al., 2023; Newhart, 2023), and promotes positive emotions and prosocial behavior (Huang et al., 2023).

### 1.2 Isolation and non-isolation group of Chinese college students

Social isolation was a fundamental strategy to deal with COVID-19 in China. Moreover, unlike other countries around the world, the Chinese lockdown measures were stringent and widespread. As for college students, they could be roughly divided into two groups during the pandemic. The non-isolation group included college students who were quarantined on campus. They were unable to leave the campus but could move around on it and still maintained basic social communication with classmates and teachers. The isolation group included students who were isolated outside the campus, not only from their previous social relationships but also in terms of communication with their classmates and teachers. Typically, these students are required to isolated for 7 days out of campus if they are suspected of being infected with COVID-19. Subsequently, they move into a designated area in campus and continue to isolating for an additional 7 days. After a cumulative 14 days period can they return to their classmates. Therefore, students who had undergone a 14 days isolate progression and then returned to campus at last were defined as the isolated group in this study.

Isolation has significantly transformed the social support networks of college students, and brought them more mental health risks. A meta-analysis showed that one in four Chinese college students experienced depressive symptoms during the pandemic (Luo et al., 2021). Another study indicates that Chinese college students during the pandemic are at a higher risk of experiencing PTSD symptoms and have difficulty accessing mental health support (Wu et al., 2025). There is also evidence that social isolation is a key factor in the triggering psychological distress among Chinese college students (Sun et al., 2023).

During isolation, college students saw a significant drop in emotional, instrumental, and informational support, which correlated positively with psychological distress (Wang et al., 2020). In contrast, non-isolated students maintained some social networks and interaction frequency, even if their support systems were partially affected by the pandemic (Huckins et al., 2020). Isolation policies disrupted traditional campus social networks, causing a severe reduction in peer support for academics and emotions (Huckins et al., 2020). While family support rose during isolation, its negative impact on overall social support perception outweighed the reduction in peer support (Copeland et al., 2021).

However, there is still limited knowledge about the mental health risks that prolonged strict lockdowns and social support bring to college students.

It is well-known that cultural factors exert a profound influence on the efficacy of social support. The inherent norms, values, and beliefs of a culture dictate the ways in which individuals engage with social support systems, thereby affecting their capacity to achieve wellbeing and resilience. Considering that a relationship-based culture is one of the characteristics of Chinese society (Ho, 1998). Collectivism was significantly associated with social support and prosocial tendencies among Chinese college students (Zhang and Han, 2023) and collectivistic values often serve as a buffer against feelings of existential isolation (Park and Pinel, 2020). Therefore, it is hoped to further explore the influence of culture may on the complex relationship between isolation and social support among Chinese college students during the pandemic.

### 1.3 Mental health and COVID-19-burnout

As the COVID-19 pandemic continues, its long-term impact is receiving increased attention. There has been a transition from a temporary shock to COVID-19 being a part of life with the continuation of the pandemic. This is particularly evident among the Chinese population, who have faced restrictions for 3 years, leading to a worsening situation. In acknowledgment of the mental health repercussions of enduring the emotional and social strain associated with the COVID-19 pandemic, a new term has been introduced: “COVID-19-burnout.” It was defined as a result of long-term sustained impact of COVID-19 (Yıldırım and Solmaz, 2022).

In general, burnout is considered a continuous psychological reaction to the relentless emotional and interpersonal stressors that are commonly encountered in the workplace (Maslach and Leiter, 2016), and is more often described as a state of restlessness that is considered a precursor to depression (Zhou et al., 2021). It was found that COVID-19-burnout was common in Turkish adults (Yıldırım and Solmaz, 2022). Previous work has demonstrated a correlation between the phenomenon of burnout, which has emerged in the context of the COVID-19 pandemic, and a range of unfavorable health sequelae, notably the exacerbation of anxiety and depressive tendencies (Koutsimani et al., 2019) and more severe learning burnout and related negative psychological emotions (Salmela-Aro et al., 2022).

Empirical evidence has linked the state of COVID-19-burnout with detrimental health consequences, such as increased anxiety and depressive symptoms (Sun et al., 2022). Burnout has shown a positive association with psychological distress, exemplified by depression, anxiety, and stress, during the COVID-19 pandemic (Talaee et al., 2020; Conejero et al., 2023). Thus, COVID-19 has exerted both short- and long-term effects. Nonetheless, relevant research is still limited; as well as examining the short-term effects of COVID-19 on university students, it also appears necessary to explore its long-term impact. By doing so, we can strengthen the preventive measures aimed at safeguarding the mental health of this group. Psychological distress, such as anxiety and depression, has been the subject of numerous studies since COVID-19 entered its third year. However, COVID-19-burnout, a long-term consequence of COVID-19, has not received much attention.

### 1.4 The present study

In summary, despite the significant amount of research on the association between social assistance and psychological health in students, there is a paucity of research on the differences between isolated and non-isolated groups of students, particularly in the Chinese context. Moreover, existing studies have emphasized the short-term mental health risks posed by COVID-19 (such as anxiety and depression), with less attention being paid to the long-term effects (such as COVID-19-burnout). This study could improve understanding of the pathways through which social support alleviates both the long- and short-term effects of COVID-19 among Chinese college students, as well as generate new ideas for proactive mental health services that could be offered to students at different times. Therefore, emotional reactions, such as psychological distress, as well as long-term consequences of COVID-19 were including all in this study. However, the focus is on the long-term consequences of COVID-19, the COVID-19-burnout.

The main effect model and the stress buffer model are the primary theories that explain the mechanism of social support (Cohen and Wills, 1985). However, there has long been a debate between them. The primary cause is the difficulty in differentiating between potential social support (such as institutions, laws, etc., main effect model) and social support mainly from individuals (stress buffer model). China’s strict epidemic control provided a rare opportunity to demonstrate the difference between social support mainly from society and that mainly from individuals. That is to say, the normal social support available to the isolated group will be significantly limited. They perceive mainly support from the government or society (main-effect model). In contrast, those who are not isolated experience more from their personal social relationships (stress buffer model). Thus, isolation would be served as a good example for distinguishing the experienced social support mainly from society and individual.

Therefore, this study attempts to explore (1) the difference between the short- and long-term risks to wellbeing posed by COVID-19 in Chinese college students; and (2) the impact of social detachment on the mental health of Chinese tertiary education students throughout the COVID-19 pandemic. Accordingly, we introduce the following hypotheses: (H1) There are significant differences in COVID-19-burnout, social support, and mental health between isolated and non-isolated groups; (H2) Social support is significantly and inversely related to psychological wellbeing and COVID-19-burnout; and (H3) Isolation moderates the relationship between social support and psychological distress.

## 2 Materials and methods

### 2.1 Participants

The sample for this study was selected from a psychology class at an east university in China from September to November in 2022. Similar to students, teachers’ behaviors were also restricted greatly during the pandemic. Therefore, this study employed a convenience sampling method, with the sample limited to the classes taught by the researchers. And so, this survey was arranged as part of course to help the students understand concepts related to psychological measurement. They were informed that the questionnaire was to be completed voluntarily, and that their semester grades would not be affected if they chose not to participate.

### 2.2 Measures

#### 2.2.1 Perceived social support scale (PSSS)

To assess social support, the Chinese version of the PSSS was applied in this study (Jiang, 2005). The scale was developed by Zimet et al. (1990) and includes 12 items distributed over two subscales (outside family and within family) rated on a seven-point Likert scale ranging from 1 (indicating strong agreement) to 7 (indicating strong disagreement). The scale score reflects the level of social support an individual receives. This scale exhibits good psychometric performance across studies. The Cronbach’s α coefficient rages from 0.78 to 0.88 (Panzeri et al., 2022; Wu et al., 2024). In this study, the Cronbach’s α coefficient was 0.92 (The fit indices was as follows: chi-square x^2^/df = 3.244, RMSEA = 0.076, RMR = 0.131, CFI = 0.963, NFI = 0.947, AGFI = 0.900, and GFI = 0.934).

#### 2.2.2 COVID-19-burnout scale (COVID-19-BS)

In this study, the COVID-19-BS (Yıldırım and Solmaz, 2022) was applied to assess burnout associated with the COVID-19 pandemic. It was adapted from the Burnout Measure-Short Version (Alves et al., 2004), which has a one-dimensional structure and includes 10 items measured on a five-point Likert scale. Responses range from 1 (“never”) to 5 (“always”). The total score serves as an indicator of the severity of the burnout experienced during the pandemic. The Cronbach’s α coefficient rages from.88 to.93 in other study (Haktanir et al., 2022; Yıldırım and Solmaz, 2022). And in this study, the Cronbach’s α = 0.90, the fit indices were as follows: chi-square x^2^/df = 2.858, RMSEA = 0.069, RMR = 0.039, CFI = 0.972, NFI = 0.958, AGFI = 0.925, and GFI = 0.959.

#### 2.2.3 Brief Symptom Inventory-18 (BSI-18)

The Brief Symptom Inventory-18 (BSI-18) (Zabora et al., 2001), a condensed adaptation of the Symptom-Checklist-90 (SCL90), was used to assess three dimensions of psychological distress: anxiety, depression, and somatization. Each of the 18 items is scored using a five-point Likert scale, with responses ranging from 0 (“not at all”) to 4 (“extremely”). The internal consistency coefficient of the scale ranges from 0.83 to 0.93 (Lancaster et al., 2016; Franke et al., 2017). In the current research, the Cronbach’s α coefficient was 0.95. However, confirmatory factor analysis shown that the fit indices were not very satisfactory in this study: chi-square x^2^/df = 6.355, RMSEA = 0.117, RMR = 0.033, CFI = 0.864, NFI = 0.843, AGFI = 0.712, and GFI = 0.784).

### 2.3 Procedure

The questionnaires were adapted into online formats using the Questionnaire Star Survey platform^1^, an online tool for data collection. The eligibility criteria for the study were being a university student, being in good health, being aged ≥ 17 years, having been enrolled in a university course for the last 3 years following the emergence of COVID-19, and having access to an electronic device. We included a clear definition of isolation in the questionnaire’s introduction to help participants better recognize their own circumstances.

Completion of the entire questionnaire takes about 15 min, including five lie detection questions. The participants were required to answer all questions. All questionnaires had to be completed during class time. In the end, 406 college students finished the survey. After removing invalid questionnaires (completed in < 200 s, more than three incorrect answers on the lie detection questions, or having duplicate or excessively similar responses), a total of 394 valid responses were obtained. The valid response rate was approximately 97.04%.

This research was sanctioned by the ethics committee of the ethics committee of the Affiliated Hospital of Nanjing University of Traditional Chinese Medicine in accordance with the Declaration of Helsinki.

### 2.4 Data analysis

Data analysis was conducted utilizing SPSS 22.0, AMOS for Windows and Process4.5. Multiple regression analysis was used to exploring the moderating effect of isolation between social support and psychological distress (Wen et al., 2008). *T*-tests and Pearson correlation analyses were also employed to examine the data. The Process 4.5 program was used to analyze the moderation effects in this study (Hayes, 2017).

## 3 Results

The demographic variables of interest in this study included gender (male/female), age, major, grade (1–4), family economic status (below average, average, above average), parental occupation [government employee, company employee, self-employed, other (e.g., unemployed)], history of COVID-19 infection (yes/no), and history of isolation (yes/no).

An online questionnaire garnered valid responses from a total of 394 students, as stated above, comprising 117 males (29.7%) and 277 females (70.3%). The age range was 17–22 years, with the mean ± standard deviation age being 19.09 ± 0.82 years. The gender distribution mirrors that of the university’s overall population. As an elective course, students were drawn from a number of academic years. There were 77 (19.5%) first-year, 227 (57.6%) second-year, 88 (22.3%) third-year, and only 2 (0.5%) fourth-year students. These students were pursuing studies in three major disciplines: Clinical Medicine Science (*n* = 120, 30.5%), Pharmacy (*n* = 121, 30.7%), and Rehabilitation (*n* = 153, 38.8%). The reported occupations of the students’ fathers were as follows: government employees (civil servants, teachers, doctors, etc.), *n* = 54 (13.7%); company employees, *n* = 154 (39.1%); self-employed, *n* = 88 (22.3%); and other occupations or unemployed, *n* = 98 (24.9%). The reported occupations of the students’ mothers were as follows: government employees, *n* = 57 (14.5%); company employees, *n* = 141 (35.8%); self-employed, *n* = 66 (16.8%); and other occupation or unemployed, *n* = 130 (33.0%). There were no self-reported cases of COVID-19 among the survey respondents; however, 8 (2.0%) students indicated that a relative had been infected with the virus. Regarding isolation status, 122 (31.0%) of the participants were identified as isolated, while the remaining 272 (69.0%) were not.

COVID-19-burnout, psychological distress—encompassing somatization, anxiety, and depression—and social support did not differ significantly (*p* > 0.05) among the students according to the demographic factors of gender, academic grade, or family economic status.

However, there were significant differences in paternal occupation according to social support (within the family) (F = 2.678, *P* = 0.047) and somatization (F = 3.146, *P* = 0.025). The least significant difference (LSD) test revealed that university students whose fathers’ occupation was “other” or unemployed had the lowest level of family social support. The LSD test of the somatization factor indicated that students whose fathers were company employees had significantly lower somatization scores compared with students whose fathers were self-employed or in the “other or unemployed” category, although the difference was not significant compared with the group having fathers in government positions. Significant differences were also found in social support (within the family) according to maternal occupation (F = 4.007, *P* = 0.008). The LSD test showed that university students whose mothers were government employees scored significantly higher in family social support compared with those in the other three groups.

The independent-samples *t*-test highlighted a statistically significant difference in COVID-19-burnout between students in isolation (*n* = 122) and those not in isolation (*n* = 272). Isolated students, regardless of the source of their social support, had a significantly diminished sense of support compared with their counterparts. The burnout levels of the isolated group were notably higher (t = 2.42, *p* < 0.01), and their psychological distress was also significantly more severe (t = 2.96, *p* < 0.01) (Table 1).

**TABLE 1:** Results of independent-samples *t*-test of isolation (*N* = 394).

Variables	Isolation M ± SD (*N* = 122)	Non-isolation M ± SD (*N* = 272)	t
Social support	60.78 ± 11.49	64.81 ± 1061	−3.396**
Social support (within the family)	20.98 ± 4.56	22.24 ± 3.88	−2.820**
Social support (outside the family)	39.80 ± 7.76	42.57 ± 7.61	−3.321**
COVID-19-burnout	26.20 ± 7.95	24.25 ± 7.09	2.420*
Psychological distress	1.84 ± 0.57	1.67 ± 0.51	2.958**
Somatization	1.80 ± 0.60	1.65 ± 0.57	2.315*
Anxiety	1.82 ± 0.64	1.65 ± 0.55	2.672**
Depression	1.90 ± 0.61	1.71 ± 0.55	3.147**

In all tables,

*p < 0.05,

**p < 0.01.

Table 2 illustrates the significant associations among variables. Social support showed a significant inverse relationship with COVID-19-burnout (r = −0.142, *p* < 0.01) and psychological distress (r = −0.356, *p* < 0.01). Within-family social support correlated negatively only with psychological distress (r = −0.314, *p* < 0.01), whereas outside-family social support was negatively associated with both COVID-19-burnout (r = −0.157, *p* < 0.01) and psychological distress (r = −0.339, *p* < 0.01). Furthermore, there was a notable positive correlation between COVID-19-burnout and psychological distress (r = 0.409, *p* < 0.01).

**TABLE 2:** Results of Pearson correlation analysis (*N* = 394).

Variable	1	2	3	4	5	6	7
1. Social support	–	–	–	–	–	–	–
2. Social support (within the family)	0.863**	–	–	–	–	–	–
3. Social support (outside the family)	0.963**	0.695**	–	–	–	–	–
4. COVID-19-burnout	−0.142**	−0.083	−0.157**	–	–	–	–
5. Psychological distress	−0.356**	−0.314**	−0.339**	0.409**	–	–	–
6. Somatization	−0.261**	−0.234**	−0.246**	0.308**	0.891**	–	–
7. Anxiety	−0.320**	−0.282**	−0.306**	0.405**	0.951**	0.779**	–
8. Depression	−0.399**	−0.350**	−0.381**	0.413**	0.910**	0.670**	0.837**

1. Social support; 2. Social support within the family (subscale of the Social Support Scale), 3. Social support outside the family (subscale of the Social Support Scale); 4. COVID-19-burnout; 5. Psychological distress (total scale score); 6. Somatization (subscale of psychological distress); 7. Anxiety (subtype of psychological distress); 8. Depression (subtype of psychological distress). In all tables,

*p < 0.05,

**p < 0.01.

**FIGURE 1:**
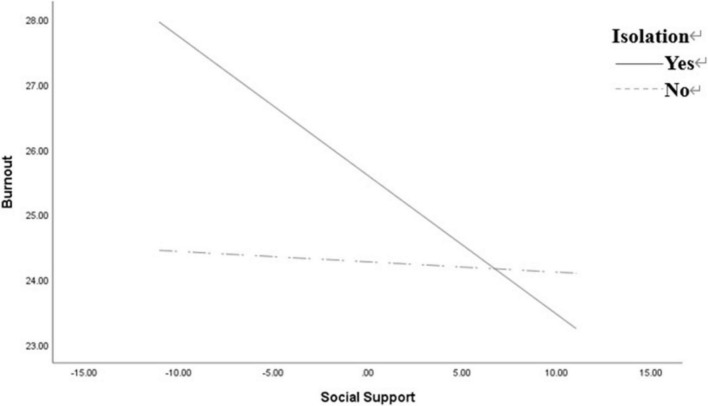
Simple slope of the moderating effect of isolation on the relationship between social support and COVID-19-burnout.

In moderation analysis with social support as the predictor, psychological symptoms as the outcome, and isolation as the potential moderator, the moderation effect was found to be non-significant (*p* = 0.26). However, when COVID-19-burnout was used as the dependent variable, the moderating effect was significant. In analysis, isolation served as the categorical moderator variable, social support functioned as the continuous predictor variable, and COVID-19-burnout was designated as the outcome variable. The results showed that isolation had a significant regulatory effect (β = −0.213, *p* < 0.05), and the interaction term was significant (β = 0.198, *p* < 0.01). Both social support and isolation were significant predictors of COVID-19-burnout, and the higher the level of social support (β = −0.411, *p* < 0.01), the lower the level of COVID-19-burnout. Simple slope analysis showed that the degree of social support had a greater positive effect on the students in the Isolated-group compared with those who were not isolated (Figure 1 and Table 3).

**TABLE 3:** Analysis of the moderating effect of isolation on the relationship between social support and COVID-19-burnout.

Model	Beta	SE	*t*	*P*	CI	
					LLCI	ULCI
Social support	−0.411	0.122	−3.364	0.001	−0.651	−0.171
Isolation	−1.329	0.809	−1.644	0.101	−2.919	−0.260
Yes	−0.213	0.057	−3.716	0.000	−0.326	−0.100
No	−0.016	0.042	−0.383	0.701	−0.098	−0.066
Interaction term	0.198	0.071	2.787	0.006	0.058	0.337

The total R^2^ of the social support sample is 0.049, and the ΔR^2^ of the interaction term is 0.019; Interaction term = Social support × isolation.

## 4 Discussion

In line with Hypothesis 1, the data presented in Table 1 reveal significant differences in social support, psychological wellbeing, and the impact of COVID-19-burnout between the two groups. The results suggest that social isolation was a risk factor for low wellbeing among the college students. Since the emergence of COVID-19, measures to isolate individuals have been widely implemented globally, and particularly in China, to curb the pandemic’s progression. However, these preventative strategies inadvertently triggered a range of psychological conditions among youths, including obsessive-compulsive tendencies, anxiety, health anxiety, melancholy, and neurasthenia (Chen et al., 2020). Research has highlighted a correlation between enforced isolation due to COVID-19 and an escalation in anxiety and depressive symptoms among university students (Huang and Liu, 2023). This correlation substantiates the assertion that social isolation poses a significant risk to the mental health of Chinese students (Yang et al., 2023). Our findings corroborate the adverse effects of social seclusion on college and university students’ mental health and psychological wellbeing, underscoring the imperative for policymakers to consider the psychological repercussions of isolation more closely when devising future public health strategies.

Second, the results provide partial support for Hypothesis 2, with the specific details outlined in Table 2. As we can see, there was a significant inverse correlation between perceived social support and the extent of psychological distress, which encompasses feelings of anxiety, depressive symptoms, and somatic complaints, as well as the burnout experienced due to the COVID-19 outbreak within the college student population. This is consistent with prior research, where a study demonstrated that social support served to mitigate the risk of anxiety and depression among college students during the lockdown period (Yin et al., 2022) and was associated with greater participation of college students in sports activities (Wang et al., 2024). Moreover, the increasing implementation of online courses has further weakened social interaction (Race, 2020) and increased the academic pressure on students (Xiao and Li, 2020). In China, diminished social support has been found to be associated with poorer mental health outcomes among college students (Liu et al., 2023). The COVID-19 pandemic has underscored the critical role of perceived social support in safeguarding the mental health of college students.

The present research provides a compelling perspective, highlighting the profound effect of culture on the efficacy of social support: while there is a clear connection between social support and COVID-19-burnout, the subscale scores suggest that support from outside the family (r = −0.157, *P* = 0.002) is more important than within-family support (r = −0.083, *P* = 0.099). This suggests that social support from outside the family is a key factor in alleviating long-term stress, including COVID-19-burnout. The pandemic has highlighted the role of collectivistic values in providing a buffer against feelings of isolation, especially among those confronted with the myriad stresses associated with these challenging times (Park and Pinel, 2020). The collectivism that characterizes Chinese society is therefore relevant in this regard: in a society that emphasizes collectivism, the determinants of future stress are more likely to be rooted in the societal collective rather than in the support provided within families. Evidence indicates that collectivism is significantly correlated with the social support and prosocial behaviors exhibited by Chinese college students (Zhang and Han, 2023). In summary, for Chinese individuals, while social support from within the family is beneficial for alleviating short-term emotional symptoms (such as anxiety, depression, and somatization), outside-family social support (mainly from the government) appears to be more crucial for mitigating long-term stress (COVID-19-burnout). However, further evidence is required to confirm these interpretations.

Third, Hypothesis 3 also received partial support. A moderating effect was observed in the relationship between social support and COVID-19-burnout, but not in the relationship between social support and mental health. Consistent with numerous prior investigations, this study’s outcomes lend credence to the beneficial effects of social support on psychological wellbeing (Li et al., 2021), especially in students during the COVID-19 period (Qi et al., 2020). However, in contrast to previous research findings, the results of this study indicate that social support did not have a significant impact on COVID-19-burnout in the non-isolation group compared with the isolation group. For instance, previous research demonstrated a negative correlation between social support and academic burnout (Ye et al., 2021), and social support was found to mediate the negative effects of burnout on health regardless of gender (Ruisoto et al., 2021).

This result can be explained by the daily lives of Chinese students. Despite COVID-19-induced restrictions on social activities, the non-isolation group’s routines remained largely consistent on campus. Since the onset of COVID-19, while campus entry and exit were strictly regulated, non-isolated students could still move freely within the campus. Consequently, regression coefficients for social support and mental/physical symptoms were significant for both groups of university students. However, as shown in Figure 1, social support levels significantly influenced COVID-19-burnout in isolated students, an effect not observed in the non-isolated group. Given that within-family social support was not significantly correlated with COVID-19-burnout (see Table 2), this suggests that in a collectivist society like China, vulnerability to COVID-19-burnout may be more influenced by broader societal factors than by familial ties.

In contrast, isolated students experienced separation from their daily interpersonal interactions with family, friends, and classmates. They were required to remain on campus or in temporary housing during suspected COVID-19 periods, which profoundly altered their access to social support. For these students, physical separation and practical challenges made online communication their primary means of obtaining within-family social support, while outside-family support (e.g., governmental and institutional assistance) became their de facto lifeline. As a result, they depended far more on external systems, such as government programs or university resources, for daily needs and well-being. Although online communication helped sustain within-family social support, it provided limited tangible assistance, leaving individuals reliant on external support to mitigate COVID-19-burnout. Data indicate that outside-family social support had a more direct and vital impact on reducing COVID-19-burnout compared to within-family support, which exerted minimal effects despite online facilitation.

Furthermore, online communication may alleviate short-term emotional issues, such as anxiety and depression, but it can exacerbate long-term concerns regarding future expectations. This heightened uncertainty and despair among isolated students may explain why isolation’s mediating effect on general mental health was not pronounced, yet it significantly amplified COVID-19-burnout. The broader impacts of COVID-19 thus appear greater for isolated students, who felt excluded or detached from society at large. This underscores the significant moderating role of isolation: amid major societal pressures like COVID-19, outside-family social support gains heightened importance relative to within-family support in addressing burnout.

These findings highlight cultural factors as crucial for designing effective psychological interventions during public health emergencies. For isolated students, strategies should be adjusted to emphasize addressing the deficiencies stemming from limited in-person within-family support, potentially incorporating enhanced external resources to bolster resilience against burnout and future-oriented distress.

Finally, the outcomes of this research suggest that the effects of social support on the transient and persistent symptoms of mental health may not be uniform. The main-effect model and stress-buffer model were introduced to help us understand the underlying mechanism of social support (Cohen and Wills, 1985). The former model emphasizes the support provided by the social environment, regardless of whether individuals are aware of it (main effect). In contrast, the latter model places greater emphasis on the role of individuals’ perceived level of social support in situations where there is awareness of stress (stress buffer). According to the stress-buffer model, social support acts as an intermediary between subjective evaluations and stressful events by reducing the perceived severity of stressful events (Alloway and Bebbington, 1987). The results of this study show that social support has a positive effect on wellbeing among students in both mental and COVID-19-burnout (see Table 2), supporting the main-effect model of social support. At the same time, this study also found that students who have experienced isolation and have low social support exhibit significantly higher levels of COVID-19-burnout (see Figure 1). This seems to align more with the buffering model of social support, but only in relation to COVID-19-burnout. In other words, during a major social stressor like COVID-19, Chinese college students generally adhere to the main-effect model of social support, although for those in the isolated group, the buffering model is more applicable. This indicates that in the face of major social stressors, the main and buffering effects of social support operate synergistically. Naturally, this also prompted us to consider whether this characteristic is related to the collectivist nature of Chinese culture. This is a question worth exploring in the future, reminding us that long-term isolation is still a potential risk factor with respect to the mental health of college students. Therefore, college students still merit attention and support during isolation to ensure that their mental health is not adversely affected.

In summary, this research yielded four principal results: (1) Isolation is a risk factor for poor mental health outcomes among college students; (2) social support has a positive effect on the mental health of college students; and (3) Within-family social support correlated negatively only with psychological distress, whereas outside-family social support was negatively associated with both COVID-19-burnout and psychological distress. (4) Isolation moderated the relationship between social support and COVID-19-burnout, but not social support and psychological symptoms.

These results suggest that interventions for mental health issues caused by the pandemic among college students require a long-term focus, particularly for those who have experienced isolation. Accordingly, consideration of cultural factors may be crucial to design effective psychological interventions for major public health emergencies. At the same time, the results of this study provide insight into the underlying mechanism of social support in circumstances of severe social stress. Finally, the results also suggest that the way in which social support functions may differ across cultures; this warrants further exploration in the future.

Several limitations of this research should be mentioned. First, the research sample was limited to students enrolled in a psychology course at a university in eastern China, along with the use of convenience sampling. This may elevate sample homogeneity, thereby undermining population representativeness and compromising external validity. These factors constrain the generalizability of the findings. And so, the high degree of homogeneity in the sample and a lack of representativeness should be considered before generalizing these findings to other groups.

Secondly, it is hard to establish causal relationships among variables by the cross-sectional design, and at the same time, effectively control confounding variables in this research. Furthermore, it also rose risks related to reverse causality and confound by other third variables.

Third, social desirability bias and recall error would be easily induced under the conditions of self-report questionnaires. These factors may lead to a negative impact on the reliability of the research results.

Fourthly, the timing of this survey is unique. The data collection for this study took place merely one month prior to the abrupt end of China’s COVID-19 lockdown policies. Although, as previously noted, this timing increases the significance of the study, it is crucial to be emphasized that it only reflects the phases one month before the conclusion of the lockdown policies and cannot be simply generalized to other phases.

Other factors, such as pre-existing mental health conditions or specific isolation durations, could influence results ether. These issues may also have a negative impact on the reliability of the study results.

Fifth, this study did not effectively control confounding variables duping to limited conditions. For example, participants’ social networking usage patterns, baseline mental health status, and the specific aspects of social support were not fully considered in the questionnaire design. These limitations may potentially impact the inferences drawn about the relationships between variables in this study. And so, it is required more careful interpretation and cautious generalization of the findings. Additionally, while the findings might have been influenced by cultural factors, further research is required to confirm this.

## Data Availability

The original contributions presented in this study are included in this article/supplementary material, further inquiries can be directed to the corresponding authors.

## Footnotes

1
https://www.wjx.cn/
